# Measuring Nurses’ and Physicians’ Attitudes and Perceptions of the Appropriate Interventions towards Intimate Partner Violence in Saudi Arabia

**DOI:** 10.3390/healthcare10081430

**Published:** 2022-07-30

**Authors:** Wafa Hamad Almegewly, Sanna Hawamdah, Fatchima Laouali Moussa, Wireen Leila Tanggawohn Dator, Anwar Alonezi, Majid Al-Eissa

**Affiliations:** 1 Department of Community Health Nursing, College of Nursing, Princess Nourah bint Abdulrahman University, Riyadh 11671, Saudi Arabia; ansalanazi@pnu.edu.sa; 2 Independent Researcher, Philadelphia, PA 19104, USA; hawamdehs@gmail.com; 3 Department of Medical-Surgical Nursing, College of Nursing, Princess Nourah bint Abdulrahman University, Riyadh 11671, Saudi Arabia; flmoussa@pnu.edu.sa (F.L.M.); wldator@pnu.edu.sa (W.L.T.D.); 4 Department of National Family Safety Program, Ministry of National Guard Health Affairs (MNGHA), King Saud bin Abdulaziz University for Health Sciences (KSAU-HS), Riyadh 11426, Saudi Arabia; eissam@ngha.med.sa; 5 Department of Pediatrics, King Abdullah International Medical Research Center (KAIMRC), Riyadh 11481, Saudi Arabia

**Keywords:** intimate partner violence, Saudi Arabia, emergency department, nurses, physicians, practice

## Abstract

Background: Intimate partner violence (IPV) is considered the most common form of violence against women worldwide, concerning public health, safety, and human rights. However, little to no studies in Saudi Arabia have explored the attitude and perception of health care providers working in emergency departments toward IPV. This study aimed to measure the attitude and perception of Emergency Room (ER) health care providers towards the appropriate intervention for IPV. Methods: This is a cross-sectional quantitative study. Data was collected from a convenient sample of nurses (*n* = 88) and physicians (*n* = 18) working in ER, using Readiness to Manage Intimate Partner Violence Survey (PREMIS). Data was collected from two hospitals in Riyadh, Saudi Arabia, and descriptive analysis was used to analyze the data. Results: The majority of the respondents were aged 18–40 (*n* = 106, 78%), while 22% were 41–60 years old, 69% were female, and 31% were male. Eighty-five percent were nurses and 15% were physicians. The majority of the respondents did not have any training on IPV and had gained knowledge or skills mostly during their medical/nursing classroom and clinical training. The analysis revealed that the participants had moderate levels of overall preparedness, knowledge about IPV, and perceived knowledge, with a mean score of 2.30, 18.62, and 2.18, respectively. The respondents had low scores in practice issues in new diagnosis (0.91), current screening (1.69), and actions when IPV is identified (0.91). The perceived preparedness and knowledge have a significant positive correlation, as shown by an r value of 0.8476 and a *p*-value of <0.05. Conclusion: The study shows that participants stated minimal previous IPV knowledge and training. It is necessary to put in place adequate resources and specific training programs to overcome this issue for both ER nurses and physicians.

## 1. Introduction

Worldwide, intimate partner violence (IPV) is considered the most common form of violence against women concerning public health, safety, and human rights. IPV is defined as “any threatening behavior within an intimate relationship that leads to physical, psychological, or sexual injury to those in the relationship” [1]. Globally, about 1 in 3 (35%) of women have been exposed either to physical and/or sexual intimate partner violence or non-partner sexual violence [2]. Among 161 countries, the results showed that approximately 27% of women aged 15–49 years have experienced physical or sexual, or both, intimate partner violence [3]. From 2004 to 2006, 713 cases of family violence and 600 cases of personal affairs were reported by the National Human Rights Society (NSHR) in Saudi Arabia [4]. In 2017, Saudi courts received 1059 cases of violence against women, including 348 cases of physical violence, 59 cases of domestic violence, and 65 cases of sexual abuse [5]. Domestic violence takes many forms, such as physical, sexual, and emotional. Physical violence includes beating, punching, kicking, threatening, and attaching by weapon [6]. Sexual violence involves forcible sexual acts or any form of sexual coercion, while psychological violence comprises continuous offenses, frustration, excessive anger, and bullying [1].

In Saudi Arabia, many cross-sectional quantitative studies have been carried out, assessing the frequency of domestic violence. In Riyadh, Saudi Arabia, 1883 married Saudi females were recutied from 18 primary health care and 13 varied settings such as teaching and governmental institutes and social welfare organizations [7]. They found that the lifetime prevalence of IPV was 43%, including 36.8% of controlling behaviors by husbands, especially if seeking permission to seek medical help, which may lead to long-term consequences of physical, psychological, and social problems. In Jeddah, Saudi Arabia, a study was conducted to identify the leading factors of IPV against 2301 women. The results illustrated that the participants were emotionally, physically, and sexually abused (29%, 11.6%, and 4.8%, respectively) [8]. Some demographical factors increase women’s risk of experiencing IPV, such as a low education level, marital status and having more children, being unemployed, and being financially dependent on their spouses. In Riyadh, a recent study found that the prevalence of IPV was 44.8%, composed of 18.5% physical abuse, 25.5% emotional abuse, 19.2% sexual abuse, and 25.3% economic abuse. Furthermore, they also found that physical violence is common among women who live with another wife in the same house [9]. These factors might be used by health care providers to detectthese hidden women who suffer in silence. In Saudi Arabia, the prevalence of IPV among 2000 women was 39.3%, comprising 35.9% mental abuse, 17.9% physical abuse, and 6.9% sexual violence [10]. Just before collecting the data in one month, 11% of the participants during their pregnancy were beaten, and 7% were kicked in the abdomen. IPV led many women to seek medical attention because they suffered from health conditions such as stress, abortion, and hemorrhage. On the other hand, 41.4% of these women had not reported the incidence to medical staff and preferred to share their issues with family members or friends. In Jeddah, 44% of 200 married women were exposed to physical violence from their husbands, and only 6.5% reported the incidents to the health care team [11]. Other reasons for not disclosing IPV may be related to women’s fear of being separated from their husbands, or sometimes women blame themselves for their partner’s behaviors [10]. In Arab communities, many women find it challenging to discuss domestic violence with health care providers because it is a culturally sensitive and private issue that the family members should handle to maintain family harmony and patriarchal norms [6,12,13,14]. These social factors hindered women’s willingness to report domestic violence to save family reputation and avoid social stigmatization.

In Saudi Arabia, the National Family Safety Program was launched in 2005 to protect and help victims of child abuse and domestic violence (DV). The program was established under the National Guard Health Affairs umbrella and offers healthcare education and training sessions to healthcare providers in collaboration with local and international partners. In addition, the Saudi Ministry of Health established the Department of Psychological and Social Health, which offers health, psychological, and social services in line with its 2030 vision, which provides many programs to enhance women’s rights. Even though Saudi Arabia has enacted legislation to protect women from assault, the government has taken many steps to address the issue of domestic violence, and the media is particularly vocal about it, Saudi women are still exposed to domestic violence. Therefore, this issue must be addressed in public and by the appropriate actions by health care providers.

A Saudi cross-sectional quantitative study was conducted on 151 dentists’ awareness of actions taken toward IPV. The results showed that dentists lacked education and training in assessing IPV, especially among male general practitioners compared to specialists, and they found it embarrassing to discuss IPV with patients [15]. However, the latter study cannot be generalized as the sample size (*n* = 150) is considered small. Other physicians considered reporting IPV beyond their scope of practice as they assumed that IPV is a mainly social and psychological issue and not medical [16]. Other health care providers blame the under-reporting of IPV by a lack of time and training in detecting IPV and the complexity of providing family care or education [17]. The latter studies did not explore health care providers working in the emergency department, who are in the ideal position and the first personnel to detect IPV and provide victims with appropriate interventions. A systematic review found that nurses were not prepared to deal with IPV cases because they lacked the proper education and skills in identifying the symptoms and communicating with the victims [18]. Previous studies show that gender, educational level, work experience, and receiving preservice IPV training were associated with high scores in the knowledge of IPV [19,20]. With the uprising prevalence of IPV as a public health concern, nurses and physicians are recognized for their vital role in providing healthcare services for IPV. They must have the knowledge, attitude, and capacity to manage these vulnerable patients.

The prevalence of IPV among women is increasing and breaking the silence around it is needed. ER healthcare providers are in a position to be the first personnel who witness IPV and provide care and social support. Therefore, they should be equipped with the knowledge and training resources to manage IPV. Assessing the current knowledge, attitudes, and practices of ER nurses and physicians will help in designing preparation programs on how to deal with and report IPV. Furthermore, it will enhance women’s safety and minimize the long-term effects of IPV on victims. The Saudi literature is overwhelmed with quantitative studies highlighting issues related to social determinants of domestic violence, and its prevalence. Despite the importance of measuring IPV’s prevalence, no Saudi study has explored the attitude and perception of health care providers in ER towards the appropriate intervention for IPV on a legal basis. For these reasons, this study was conducted to measure the attitude and perception of ER health care providers towards the appropriate intervention for IPV.

## 2. Materials and Methods

This is a cross-sectional quantitative study that helps to move beyond explanations of opinions, experiences, and meanings to measure nurses’ and physicians’ knowledge and preparedness to manage IPV patients. After obtaining the ethical approvals, a convenient sample of participants working in ER was recruited from two large hospitals in Riyadh. One is a teaching hospital, and the other is a military hospital that provides primary, secondary, and tertiary care. Data were collected from 17 August 2020 to December 2021. The inclusion criteria include physicians and nurses working in ER only; medical or nursing students and internal social workers were excluded.

An invitation email was sent to all nurses and physicians in the selected hospitals via medical and nursing units, with a total of (*N* = 370 nurses, *N* = 105 physicians). The target minimum sample size was (*n* = 213), calculated according to Epi info calculation with a margin of error of 5% and a confidence level of 95%. However, the collected sample size was (*n* = 106) with a low response rate of 22%.

### 2.1. Data collection Measurement and Process

Nurses and physicians were invited to complete the Readiness to Manage Intimate Partner Violence Survey (PREMIS). PREMIS is a questionnaire developed and validated in the US [21]. It comprises five sections: responder profile, background (including perceived preparedness and knowledge), actual knowledge, opinions, and practice issues. PREMIS has been used recently in a quantitative study in Saudi Arabia to assess nurses’ knowledge, attitudes, and practices towards IPV in two hospitals in Riyadh [22].

Minor changes to the questionnaire were implemented for its use in Saudi Arabia due to some technical terms not being relevant to a Saudi setting which were either changed or deleted from the profile page. For example, adding nurses as a target population besides physicians and deleting the state of practice and security number. The tool was content validated to match the Saudi context and with an overall Cronbach’s alpha of 0.7, which indicates acceptable internal consistency.

After creating an online survey link on Google Forms, it was distributed to all the target participants’ email addresses by the selected ER unit and research unit at the selected hospitals. A reminder system was set to send two reminders per month to selected hospitals, and participants were asked to complete the questionnaire during their on/off duty. The authors also asked the gatekeepers at the selected hospitals to collect the data on-site manually. The researchers visited the ER four times to distribute 104 hard copy questionnaires; only 68 filled them in. There was 36 incomplete questionnaire that was excluded because the missing data percentage exceeded 20%.

### 2.2. Ethical Considerations

Ethical approvals from the selected hospitals (ref No. 19-0194) and (ref IRBC/2212/20) were granted. The research team explained the aim and nature of the study to the ER department heads. The informed consent was obtained electronically on the first page of the online/printed questionnaire from the target participants who agreed to participate voluntarily.

### 2.3. Data Analysis

Descriptive statistical analysis was performed using the Statistical Package for Social Sciences (SPSS) version 24. The relationship between background and opinion scale on IPV was measured using Pearson’s correlation coefficient, and it was accepted if 𝑃 was less than or equal to 0.05.

## 3. Results

### 3.1. Demographic and Work Profiles

The majority of the respondents were aged 18–40 (*n* = 106, 78%), while 22% were 41–60 years old, 69% were female, and 31% were male. Eighty-five percent were nurses, and 15% were physicians. There were 37% among them with 6–10 years’ work experience, 20% with 2–5 years, 18% with 11–15 years, 17% with 16–20 years and above, and 8% with less than two years of work experience. Nearly half of them (41.7%) took care of more than 60 patients per week, 28% had an average of 20 to 39 patients, 14.6 had 40–59, 11.7% had less than 20 patients, and 4% did not tend to patients (Table 1).

### 3.2. Previous Training on IPV

The majority (58%) of the respondents did not have any training on IPV. There were 26% who had gained knowledge or skills mostly during their medical/nursing classroom and clinical training, 4.7% during their residency/fellowship or other post-graduate training, and another 7.5% from CME programs. There were 20% who read their institution’s protocol, 12% watched a video, 11% attended a lecture/talk, 6% attended a skills training workshop, and 1% had in-depth training (Table 2).

### 3.3. Background on IPV: Perceived Preparedness

The preparedness level of the respondents was moderate, as shown by a mean score of 2.30. The scores showed that the respondents who had the highest level of preparedness on the scale appropriately responded to disclosures of abuse (2.42), followed by identifying IPV indicators based on patient history and physical examination (2.41), documenting IPV history and physical examination findings on the patient’s chart (2.31), and helping the IPV victim assess his/her danger of lethality (2.24). On the other hand, they were the least prepared on the following scales: asking appropriate questions about IPV, with a mean score of 2.10; assessing an IPV victim’s readiness to change (2.10); fulfilling state reporting requirements for IPV (2.11), and conducting a safety assessment for the victim’s children (2.12) (Table 3).

### 3.4. Perceived Knowledge

The mean score of the perceived knowledge of the respondents was 2.18, which is moderate. Their highest scores on perceived knowledge were on signs or symptoms of IPV with a mean score of 2.45, followed by determining danger for a patient experiencing IPV with a mean score of 2.28; their role in detecting IPV with 2.27; and how to document IPV on a patient’s chart with 2.25. They perceived themselves to be least knowledgeable on the following scales: the relationship between IPV and pregnancy with a mean score of 2.08; perpetrators of IPV with a mean score of 2.10; legal reporting requirements for IPV and recognizing the childhood effects of witnessing IPV both with mean scores of 2.13 (Table 4).

### 3.5. Actual Knowledge

The level of the respondents’ actual knowledge about IPV was moderate, as shown by the score of 18.62. The coverage of the knowledge of IPV that was assessed included what they know about the risk factors for becoming a victim of IPV, batterers, warning signs, reasons of an IPV victim for not leaving a violent relationship, the most appropriate ways to ask about IPV, stages of change, and general concepts (Table 5).

### 3.6. Correlation between the Perceived Preparedness, Perceived Knowledge, and Actual Knowledge

Perceived preparedness and perceived knowledge have a positive significant correlation as shown by an r value of 0.8476 and a *p*-value which is less than 0.05. The actual knowledge is shown to have a positive significant correlation with both perceived preparedness and perceived knowledge with r values of 0.9378 and 0.9305 respectively and *p*-values less than 0.05 (Table 6).

### 3.7. Opinion

The respondents from the selected hospitals agreed in their opinions of IPV in terms of alcohol/drugs, victim understanding, victim autonomy, and constraints; while they disagreed with the staff preparation, workplace issues, and self-efficacy (Table 7).

### 3.8. Practice Issues

The respondents hadlow scores in practice issues in general. They scored very low in new diagnosis (0.91), current screening (1.69), actions when IPV identified (0.91); and low in policies (2.05), activities identified IPV victim (2.52), and questioning in specific situations (2.59). They had good scores in terms of the materials available (3.44), materials given (3.56), and referral resources on site (3.56). They scored better in terms of knowledge of referral resources in the community (4.17), legal reports (4.14), and protocols (4.11) (Table 8).

### 3.9. Correlation between Background and Opinion Scales on IPV

The results from Table 9 show that opinions on preparation and legal requirements, have technically weak positive correlations, while workplace issues have moderate correlations with the amount of prior training on IPV (r = 0.1978, 0.2079, 0.30), perceived preparedness (r = 0.1769, 0.5297, 0.5468), and perceived knowledge (r = 0.1597, 0.6179, 0.6012) and the results are significant with corresponding *p*-values less than 0.05. Preparation, legal requirements, and workplace issues are also found to have a weak negative correlation with the actual knowledge about IPV (r = −0.1006, −0.1001, −0.0499) and the results are found to be not significant as the *p*-values are greater than 0.05.

Self-efficacy is found to have a weak positive correlation with the amount of training on IPV (r = 0.0353) but the result is found to be not significant as the *p*-value is greater than 0.05; moderate positive correlations were found with perceived preparedness (r = 0.5845) and perceived knowledge (r = 0.6012), and the results are significant; there was a weak negative correlation with actual knowledge (r = −0.02) but the result is not significant at 0.05.

Alcohol/drugs are found to have a weak negative correlation with the amount of prior training (r = −0.0581) and the result is not significant; weak positive correlations with the perceived preparedness (r = 0.0295) and perceived knowledge (r = 0.0246) but results are not significant; and weak positive correlation to actual knowledge (r = 0.1987) and the result is significant.

Victim understanding has weak positive correlations with the amount of prior training (r = 0.0239) and the actual knowledge (r = 0.0848) with results that are not significant, and on the other hand, has weak negative correlations with perceived preparedness (r = −0.314) and perceived knowledge (r = −0.341) and the results are significant.

Victim autonomy is found to have weak positive correlations to the amount of training (r = 0.0112), and actual knowledge (r = 0.179) with non-significant results while having weak positive correlations with perceived preparedness (r = 0.2695) and perceived knowledge (r = 0.3557) with significant results.

Constraints is found to have weak positive correlations to the amount of training (r = 0.1039), perceived preparedness (r = 0.0212), and actual knowledge (r = 0.1121); and weak negative correlation to perceived knowledge (r = −0.1233), and all with results are not significant at 0.05 (Table 9).

## 4. Discussion

This study set out with the aim of measuring the best practices in terms of training, knowledge, opinion, attitude, and clinical practice performed or perceived by health care providers (ER nurses and physicians) related to managing IPV. The results showed that (58%) of the participants had not trained enough to manage IPV, as only (20%) of them reported reading their institution’s protocol within their hospitals, and (26%) had gained knowledge as part of the undergraduate medical/nursing curriculum. The current level of previous training was low compared to other studies in which the sample were healthcare providers [19,22,23], and higher than a sample of healthcare students [24,25]. These results draw attention to the need for educational training resources, health policies, and public advocacies that support the health care providers in building their competency in dealing with IPV cases in ER. Nurses with training sessions that last more than 10 h and recently attended within the last 5 years showed more preparedness in managing IPV than others [26].

In terms of the participants’ background on IPV, the results showed that the participants were moderately prepared to respond to disclosure of abuse and had low scores in asking questions about IPV. Many studies found that health care providers, including physicians and nurses were not fully prepared to handle IPV cases [19,20,22], specifically in terms of male healthcare providers feeling uncomfortable asking sensitive questions to women [23]. Unfortunately, patients will be impacted by this, as many studies found that patients were afraid of being negatively judged and felt hesitant to express their experience of IPV to health care providers because of the latter’s inappropriate way of asking questions. Thus, women were not openly disclosing abuse unless they were directly asked [27]. It is worth noting that participants showed more preparedness in managing child abuse than IPV. The findings observed in this study mirror those of previous studies that have examined health care practitioners’ interest in child abuse more than IPV [28] which might be culturally perceived as a private family issue [29,30,31]. These results highlight the need for more educational programs for healthcare providers in communication and competency assessment tools related to IPV [32,33]. 

In regard to the perceived knowledge, participants were informed about the signs and symptoms of IPV. On the other hand, the participants reported a low level of knowledge about the relationship between IPV and pregnancy, which is considered to be an important factor in IPV [34,35]. This study showed that the overall mean of actual knowledge was low (18.62), and it is lower than a Saudi study with a mean score of (30.24) [22]. The low level of actual knowledge was also translated in the participants’ responses when they reported a lack of information about the risk factors for becoming a victim of IPV. Furthermore, the participants perceived that IPV occurs mainly because the victim cannot leave the violent relationship. Although many interventions were made to increase the level of knowledge of nurses about IPV [36,37], still it was found that both perceived and actual knowledge about IPV were low, and this is congruent with previous studies [22,25,38]. This reflects on participants’ confidence in their practical skills in managing IPV. It was reported in many studies that the actual and perceived knowledge was strongly associated with health care practice [23,39,40].

The participants’ opinions referred to their unreadiness to manage IPV due to time constraints. This might be explained by the nature of their working area which is characterized by time pressure and stress, and that is what makes ER healthcare providers differ from any other specialty. In congruence with this result, previous studies reported barriers including lack of knowledge, limited time, fear of offending, and feeling of discomfort [41,42,43,44]. Furthermore, it was found that IPV impacted negatively on the psychological well-being of ER nurses who take care of patients with IPV as they reported feelings of being overwhelmed by the nature of their work [44]. This is inconsistent with other studies that found that nurses working in departments other than ER, had reported having time to support patients with IPV [22,26]. Furthermore, both nurses’ and physicians’ readiness to address IPV was strongly associated with their personal experiences or beliefs. This was reflected in the participants’ responses to different stages of change or readiness to manage IPV, as they expressed unreadiness to change their beliefs or action about IPV.

In terms of the practical issues related to IPV, the participants reported high readiness in offering information on the referral resources located in the community to their clients. This is matched by Singhal et al. (2021), who found that women’s willingness to complete a safety plan will increase if they were referred to an IPV health care advocate and obtained access to IPV information [45]. On the other hand, the current study indicates that participants have difficulty in performing IPV diagnoses. In the emergency department, women who presented with signs of abuse-like trauma were more likely to be screened for IPV than patients with mental problems [39,46]. Acute care departments, specifically ER, are more likely to be exposed to cases of IPV, and are the front line of IPV care [45,47], thus they should be well equipped to diagnose and assess cases of IPV. Additionally, since the current data was collected during COVID-19, worldwide there were high reported rates of IPV during that period [48,49]. This might be related to physical distancing, and difficulties in accessing resources [50]. This further supports the Institute of Medicine (IOM) recommendation to involve IPV screening as part of a preventative care assessment plan. The emergency department is well placed to be an opportunity for healthcare providers to screen and detect IPV [39].Despite this, developing a universal IPV screening program that identifies the high rate of IPV is challenging in the emergency room and needs effort by the healthcare system [32,40,47].

Correlations between the the ‘background’ and ‘opinions’ subscales were significant but weak. A moderate correlation was found between the ‘workplace’ and ‘self-efficacy’ subscales which were higher than those found in all previous studies [51,52,53]. In the original version of Short et al. (2006), there were significant but small correlations between ‘actual knowledge’ and ‘perceived knowledge’, and between ‘actual knowledge’ and “opinions” subscales, except for the ‘preparation’ and ‘legal requirements’ subscales [21].

### 4.1. Strengths and Limitations

As far as we know, this is the first study in Saudi Arabia that measure ER nurses’ and physicians’ appropriate interventions in terms of training, knowledge, opinion, attitude, and clinical practice related to IPV.

In terms of limitations, this study has not been able to show the differences in managing IPV between nurses and physicians. This is because of the low response rate in general, especially from the physicians’ side. Furthermore, the low response rate was due to collecting the data during the peak of COVID-19, when all the target participants were at the front line and extremely overwhelmed. The questionnaire used was long and required more time to complete either electronically or in person, therefore, some questionnaires were not fully completed. Additionally, the number of visits to the ER department for our gatekeepers was minimized to reduce the risk of getting COVID-19. On top of that, filling in and collecting the printed questionaries was strictly limited due to COVID-19 precautions on using shared items.

A large proportion of the sample was nurses, whereas, doctors may not have sufficient statistical power to measure their readiness to screen and manage IPV; further research that recruits ER healthcare providers, particularly doctors, is required to promote diversity of responses. The refusal of doctors to participate in research has also been linked to a lack of activity or interest in the research condition [54]. In addition, more than two-thirds of the sample were women and as women, these participants were clearly more concerned about the subject, which may have influenced their responses. Furthermore, the small number of participants who were doctors allowed few comparisons of ER clinician perceptions. As a result, we are unable to highlight the source of disparities between doctors and nurses, which could be due to fundamental variations in their professional responsibilities. Further research studies in a more representative sample of ER clinicians and in other general practice settings in Saudi Arabia would be required to confirm our findings. Using qualitative design will add more insight into the lived experiences, and perceived challenges from social and cultural perspectives. However, it was difficult to run face-to-face interviews because of COVID-19 strict protective measurements that involved wearing a mask and social distancing. Due to the sensitivity of the topic, some healthcare providers may hesitate to participate in recorded interviews.

The length of the PREMIS tool was another potential limitation of this study. Responder tiredness may have influenced their response choices, particularly when answering the later items [55]. 

### 4.2. Implications for Healthcare

This study aimed to measure selected ER healthcare providers’ current levels of IPV knowledge, attitudes, and clinical practices in Saudi Arabia. It reveals persistently poor practice in training at undergraduate and postgraduate levels. Thus, the creation of specific IPV education and training programs for ER clinicians, teaching how to recognize and respond to IPV is a priority, as well as assessing the effectiveness of these educational interventions with reliable outcomes. An explicit referral path to specialized domestic violence services for women is also urgently needed. Further qualitative research is recommended to explore ER health care professionals’ experiences and perceptions of IPV.

## 5. Conclusions

Intimate partner violence remains a major global health and clinical issue with a poor health care response. We elicited ER clinicians’ levels of knowledge, attitude, and practices in the identification and management of patients experiencing IPV in Saudi Arabia. Participants stated minimal previous IPV knowledge and training and expressed a generally positive attitude towards this health issue. They performed poorly at asking IPV-related questions, identifing cases, and taking appropriate action when a case was identified. Yet, they were better prepared for making referrals and having adequate knowledge of local IPV resources.

More comprehensive and specific training on assessment and intervention for ER clinicians, including the availability of local IPV resources, is needed for both doctors and practice nurses. The PREMIS could be used to assess knowledge, attitudes, and practices in order to identify needs that could be addressed during an IPV education program. This is a reliable and simple tool for determining ER healthcare providers’ barriers to IPV screening and management, as well as focusing on specific knowledge gaps in order to build IPV education programs.

## Figures and Tables

**Table 1:** Frequency and percentage distribution for the socio-demographic profile of respondents.

	Frequency	Percent
**Age**		
18–40	82	78%
41–60	24	22%
**Gender**		
Male	36	31%
Female	70	69%
**Profession**		
Nurse	88	85%
Physician	18	15%
**Years of experience**		
<2 years	9	8.0%
2 to 5 years	22	20%
6 to 10 years	39	37%
11–15 years	19	18%
16 to >20 years	18	17%
**Average number of patients cared for per week**		
Not seeing patient	4	4.0%
Less than 20	13	11.7%
20–39	30	28.0%
40–59	16	14.6%
60 or more	43	41.7%

*n* = 106.

**Table 2:** Frequency and Percentage Distribution of Respondents who Attended Previous Training on IPV.

Training	Attendance	
	f	%
None	61	58
Read my institution’s protocol	21	20
Watched a video	13	12
Attended a lecture or talk	12	11
Attended skills-based training or workshop	6	6
Medical/nursing/other school classroom training	14	13
Medical/nursing/other school clinical training	14	13
Residency/fellowship/other post-grad training	5	4.7
CME program	8	7.5
Other in-depth training (more than 4 h)	1	1

**Table 3:** Perceived Preparedness.

Preparedness Scales	Mean	Level
a. Ask appropriate questions about IPV	2.10	Moderate
b. Appropriately respond to disclosures of abuse	2.42	Moderate
c. Identify IPV indicators based on patient history and physical examination	2.41	Moderate
d. Assess an IPV victim’s readiness to change	2.10	Moderate
e. Help an IPV victim assess his/her danger of lethality	2.24	Moderate
f. Conduct a safety assessment for the victim’s children	2.12	Moderate
g. Help an IPV victim create a safety plan	2.16	Moderate
h. Document IPV history and physical examination findings on patient’s chart	2.31	Moderate
i. Make appropriate referrals for IPV	2.19	Moderate
j. Fulfill state reporting requirements for:		
-IPV	2.11	Moderate
-Child abuse	2.20	Moderate
-Elder abuse	2.14	Moderate
Overall	2.30	Moderate

*n* = 106.

**Table 4:** Mean Distribution and Level of Perceived Knowledge of IPV.

Knowledge Scales	Mean Score	Level
a. Your legal reporting requirements for:		
-IPV	2.13	Moderate
-Child abuse	2.17	Moderate
-Elder abuse	2.15	Moderate
b. Signs or symptoms of IPV	2.45	Moderate
c. How to document IPV on patient’s chart	2.25	Moderate
d. Referral sources for IPV victims	2.14	Moderate
e. Perpetrators of IPV	2.10	Moderate
f. Relationship between IPV and pregnancy	2.08	Moderate
g. Recognizing the childhood effects of witnessing IPV	2.13	Moderate
h. What questions to ask to identify IPV	2.16	Moderate
i. Why a victim might not disclose IPV	2.21	Moderate
j. Your role in detecting IPV	2.27	Moderate
k. What to say and not say in IPV situations with a patient	2.19	Moderate
l. Determining danger for a patient experiencing IPV	2.28	Moderate
m. Developing a safety plan with an IPV victim	2.16	Moderate
n. The stages an IPV victim experiences in understanding and changing his/her situation	2.14	Moderate
Overall	2.18	Moderate

*n* = 106.

**Table 5:** Mean Distribution and Level of Actual Knowledge on IPV.

Knowledge Scales	Mean
1. Risk factors for becoming a victim of IPV	0.30
2. About batterers?	0.40
3. Warning signs	2.76
4. Reasons for an IPV victim not leaving a violent relationship	3.38
5. The most appropriate ways to ask about IPV	2.28
6. Generally true?	2.93
7. Stages of Change	2.12
8. General concepts	4.45
Overall	18.62

*n* = 106.

**Table 6:** Correlation between Perceived Preparedness, Perceived Knowledge, and Actual Knowledge of IPV.

Background Scales	Perceived Preparedness	Perceived Knowledge	Actual Knowledge
Perceived Preparedness			
Perceived Knowledge	0.8476 *		
Actual Knowledge	0.9378 *	0.9305 *	

The correlation is significant at *. * The p-value is <0.01; p < 0.05 (two-tailed); Background Scales.

**Table 7:** Mean Distribution and Agreement with Opinion on IPV.

Opinion Scales	Mean	Level
1. Staff preparation	2.46	disagree
2. Legal requirements	2.50	disagree
3. Workplace Issues	2.41	disagree
4. Self-Efficacy	2.23	disagree
5. Alcohol/Drugs	2.62	agree
6. Victim Understanding	2.58	agree
7. Victim Autonomy	2.63	agree
8. Constraints	2.92	agree
Overall	2.54	agree

*n* = 106.

**Table 8:** Mean Score Distribution on Practice issues.

Practice Issues	Mean
1. New Diagnosis	0.91
2. Current Screening	1.69
3. Questioning in Specific Situations	2.59
4. Actions when IPV Identified	0.91
5. Protocols	4.11
6. Policies	2.05
7. Legal Report	4.14
8. Activities Identified IPV Victim	2.52
9. Materials Available	3.44
10. Materials Given	3.46
11. Referral Resources on Site	4.17
12. Knowledge of Referral Resources in the Community	4.14
**Overall score**	27.97

*n* = 106.

**Table 9:** Correlation between Background and Opinion Scale on IPV.

	Background Scales			
Opinion Scales	Amount of Prior Training	Perceived Preparation	Perceived Knowledge	Actual Knowledge
Staff Preparation	0.1978 *	0.1769 *	0.1597 *	−0.1006
Legal Requirements	0.2079 *	0.5297 **	0.6179 **	−0.1001
Workplace Issues	0.30 **	0.5468 **	0.6012 **	−0.0499
Self-Efficacy	0.0353	0.5845 **	0.6012 **	−0.02
Alcohol/Drugs	−0.0581	0.0295	0.0246	0.1987 *
Victim Understanding	0.0239	−0.314 **	−0.341 **	0.0848
Victim Autonomy	0.0112	0.2695 **	0.3557 **	0.179
Constraints	0.1039	0.0212	−0.1233	0.1121

Correlation is significant ** p < 0.01 (two-tailed); * p < 0.05; N = 106; Opinion scales: Background.

## Data Availability

Not applicable.
